# Glial syncytial control fields and the low-frequency resonome of the human brain

**DOI:** 10.3389/fnhum.2026.1896291

**Published:** 2026-08-11

**Authors:** Andreu Ballús Santacana

**Affiliations:** Department of Philosophy, Universitat Autònoma de Barcelona, Barcelona, Spain

**Keywords:** active inference, astrocytes, consciousness, delta, electromagnetic field, glial syncytium, glia-neuron interaction, infra-slow oscillations

## Abstract

The low-frequency resonome of the human brain, understood as the structured hierarchy of infra-slow, delta, and theta electromagnetic field organization across approximately 0.1–8 Hz, is central to large-scale neural coordination, state transitions, sleep-wake dynamics, predictive regulation, and conscious access. Existing theories explain many aspects of these phenomena through neuronal synchronization, structural connectivity, neural-field dynamics, and recurrent cortical loops. Yet the brain also appears to require slow, spatially extended control variables integrating activity across seconds, centimeters, metabolic regimes, and neuromodulatory states, while measurable fields are generated primarily by neuronal currents. This article proposes that astrocytic syncytia provide this substrate. I introduce **glial syncytial control fields**: effective mesoscale fields arising from astrocytic syncytial organization, gap-junction coupling, calcium/IP3 signaling, extracellular ionic regulation, metabolic support, and neuromodulator-sensitive astrocyte-neuron interactions. These fields are not proposed as dominant direct generators of EEG or MEG signals. Rather, they constrain the neuronal resonome by modulating excitability, gain, damping, local time constants, synaptic plasticity thresholds, and coherence persistence. The central claim is not that astrocytes replace neurons, but that neuronal field-organization is partly shaped by a slower glial control geometry. A compact mathematical formulation is developed. A glial control field *u*_*g*_(*x, t*) obeys an effective damped-field ansatz driven by neuronal forcing and physiological fluctuations. Its low spatial modes define persistent control components that can bias neuronal low-frequency field variables ψ_*LF*_(*x, t*). A state-control formulation relates glial parameters to neuromodulatory variables, allowing the same anatomy to support different resonome geometries across arousal, sleep, attention, pathology, and development. The framework is related to neural-field and connectome models, predictive processing, active inference, electromagnetic and resonance theories of consciousness, and global workspace approaches. The hypothesis generates falsifiable predictions. Astrocyte-specific perturbations should alter slow cortical synchronization and traveling-wave persistence; gap-junction disruption should fragment long-range coherence; astrocyte-related disorders should change damping and low-dimensional geometry; and source-space phase analyses should reveal smoother, more persistent low-mode organization than phase-randomized or neuron-only null models predict. The low-frequency resonome should therefore be understood as a coupled glio-neural architecture: neurons generate primary measurable activity, while astrocytic syncytia may regulate the slow landscape within which large-scale resonance becomes stable, metastable, or pathological.

## 1 Introduction

Large-scale brain activity is not a collection of independent local computations. Neural activity is organized by slow fluctuations, traveling waves, phase gradients, metastable states, and recurrent transitions among globally coordinated regimes. Infra-slow activity, delta rhythms, and theta dynamics interact with sleep-wake state, attention, arousal, memory consolidation, predictive coordination, and conscious access (Buzsaki, 2006; Varela et al., 2001; Palva and Palva, 2012; Muller et al., 2018). The low-frequency electromagnetic field organization measured by EEG, MEG, ECoG, and intracranial recordings is therefore one of the principal forms in which brain-wide coordination becomes empirically visible.

In this article I use the term **low-frequency resonome** to denote the structured hierarchy of interacting low-frequency electromagnetic patterns and modes expressed by the brain in the infra-slow, delta, and theta ranges. The term is used in a deliberately operational sense. It does not presuppose that cognition or consciousness is identical to electromagnetic fields, nor that all relevant neural dynamics are oscillatory. It denotes the measurable organization of low-frequency electromagnetic activity as a dynamical object: a hierarchy of modes, phase relations, traveling waves, metastable transitions, and state-dependent resonance structures that constrain and express large-scale brain function.

Neuron-centered frameworks have explained much of this organization. Local excitatory-inhibitory circuits generate rhythmic activity; long-range axonal tracts coordinate distributed regions; connectome geometry shapes global modes; and neural-field equations describe propagation across cortical sheets (Buzsaki, 2006; Wang, 2010; Atasoy et al., 2016; Muller et al., 2018). Cortical traveling waves organize activity across sensory, motor, cognitive, and resting-state conditions (Muller et al., 2018), and human recordings demonstrate theta and alpha traveling waves in the neocortex (Zhang et al., 2018). Resting-state electrophysiology and fMRI reveal coherent intrinsic networks and scale-free low-frequency fluctuations (Raichle et al., 2001; Fox et al., 2005; Mantini et al., 2007; He et al., 2010; Palva and Palva, 2012). These findings justify treating the low-frequency resonome as a serious target of theory.

Yet a residual problem remains. Many low-frequency phenomena exhibit temporal integration over seconds, spatial coherence over large tissue domains, sensitivity to arousal and metabolic state, and pathological alteration in disorders involving glial dysfunction. Fast neuronal circuits can generate oscillations, and structural connectivity can constrain them, but neither alone naturally supplies a slow, spatially extended, metabolically sensitive control substrate capable of continuously tuning the conditions under which low-frequency electromagnetic organization stabilizes. This is the explanatory niche addressed by the present hypothesis.

Astrocytes possess precisely the properties required by such a control substrate. They form gap-junction-coupled syncytial networks (Giaume et al., 2010; Ma et al., 2016), tile neural tissue in partially overlapping or domain-like territories (Bushong et al., 2002; Oberheim et al., 2009), regulate extracellular potassium and neurotransmitter concentrations (Perea et al., 2009; Giaume et al., 2010), modulate synaptic gain and plasticity (Araque et al., 2014; Min and Nevian, 2012; Pannasch et al., 2011), interact with neuromodulatory systems (Poskanzer and Yuste, 2016; Reitman et al., 2023), support metabolic and neurovascular processes (Giaume et al., 2010; Clasadonte et al., 2017), and operate on slower timescales than fast synaptic transmission (Volterra et al., 2014). Astrocytic signaling is not merely local. Coupled astrocyte networks support spatially extended calcium and IP3 dynamics, and modeling work has shown that network topology and gap-junction properties strongly shape astrocytic propagation and synchrony (Goldberg et al., 2010; Lallouette et al., 2014; Verisokin et al., 2021). Astrocytes also participate causally in cortical state regulation: astrocytic activity can precede and influence state transitions, and astrocytic neuromodulatory signaling participates in cortical resynchronization (Poskanzer and Yuste, 2016; Reitman et al., 2023). Astrocyte-specific connexin disruption alters sleep-wake organization (Clasadonte et al., 2017). These facts motivate a glia-inclusive theory of the low-frequency resonome.

The novelty of the present proposal is not the general claim that astrocytes influence oscillations, synchrony, plasticity, sleep, or cortical state. That claim is already supported by experimental and modeling work. The distinct contribution is to treat astrocytic syncytia as an effective mesoscale control layer whose state can be represented by distributed variables acting on neuronal damping, gain, coupling, precision weighting, and coherence persistence. The hypothesis therefore does not merely add astrocytes as another cellular participant in rhythm generation. It proposes a control-theoretic role for syncytial astrocytic organization in shaping the geometry, stability, and state-dependence of the low-frequency neuronal resonome.

The central proposal is that astrocytic syncytia give rise to **glial syncytial control fields**. These fields are effective, mesoscale variables: they summarize the slow state of astrocytic syncytial organization and its influence on neuronal excitability, damping, coupling, and gain. They should not be identified with a literal microscopic calcium wave moving across centimeters, nor with a primary electromagnetic field generated by astrocytes themselves. The measurable EEG/MEG field remains primarily neuronal in its electrical generation. The glial contribution is instead one of control geometry. Astrocytic syncytia may help determine which neuronal electromagnetic modes are amplified, suppressed, stabilized, desynchronized, or made metastable.

Operationally, a control field is defined here as a distributed physiological variable whose state systematically modifies neuronal gain, damping, coupling, precision weighting, or dynamical stability over timescales longer than the dominant oscillatory dynamics being regulated.

**Figure 1:**
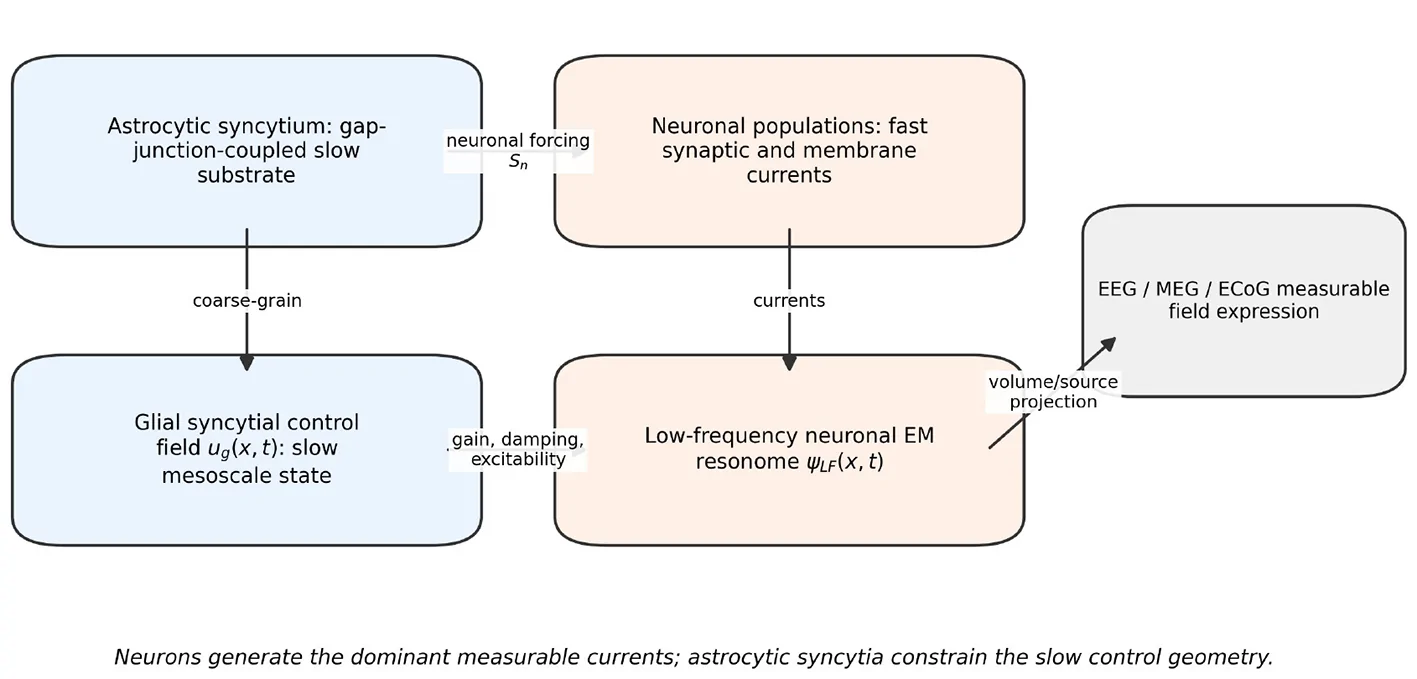
Control architecture for glial syncytial fields. Astrocytic syncytia are proposed to generate slow effective control fields that constrain, but do not directly replace, neuronal electromagnetic generation. Neuronal populations remain the dominant source of measurable EEG/MEG/ECoG currents. The glial layer modulates the damping, gain, excitability, coupling, and state-dependent stability of the low-frequency neuronal resonome.

The overall architecture is summarized in Figure 1.

This distinction is crucial. A biologically defensible glial theory of the resonome need not claim that astrocytic calcium waves directly generate delta or theta EEG rhythms. Microscopic astrocytic calcium dynamics are heterogeneous and are not proposed here as the direct carrier of macroscopic 4–8 Hz electromagnetic currents over centimeters. The stronger thesis is multiscale: slow astrocytic fields constrain neuronal electromagnetic dynamics by modulating the parameters of the neuronal system. This does not exclude intrinsic astrocytic oscillatory activity in slow ranges, nor astrocytic modulation of delta or theta dynamics. It states that, in the present model, astrocytes need not be the dominant electrical oscillator to shape the oscillatory landscape; they may function as state-dependent control fields.

The aim of this paper is therefore conceptual and theoretical. It develops the physiological rationale, minimal mathematics, and empirical predictions of glial syncytial control fields. A separate technical modeling article should develop the full Syncytial Mesh Model, including detailed graph-to-field derivations, numerical simulations, parameter inference, null-model comparison, and empirical validation. The present paper is deliberately narrower: it offers a mathematically disciplined conceptual architecture for integrating astrocytic syncytia into the theory of the low-frequency resonome.

## 2 The low-frequency resonome as a control problem

The low-frequency resonome is not simply a set of spectral peaks. It is a structured dynamical object. Its organization includes spatial phase gradients, traveling waves, cross-frequency interactions, transitions between desynchronized and synchronized regimes, infra-slow modulation of faster rhythms, and state-dependent reconfiguration across sleep, wakefulness, anesthesia, attention, and pathology (Varela et al., 2001; Buzsaki, 2006; He et al., 2010; Canolty and Knight, 2010; Palva and Palva, 2012; Muller et al., 2018; Zhang et al., 2018). Treating the resonome merely as a spectrum loses precisely what makes it biologically significant: it is a geometry of control.

A useful starting point is to distinguish three levels. First, there are **current generators**: the transmembrane and synaptic currents, especially in aligned pyramidal neuronal populations, that dominate EEG and MEG signals. Second, there are **expressed field patterns**: the measurable low-frequency electromagnetic organization produced by these currents after volume conduction, source geometry, tissue filtering, and measurement constraints. Third, there are **control variables**: slowly varying physiological states that alter the excitability, coupling, damping, and stability of the current generators. Standard EEG/MEG theory is strongest at the first two levels. The present proposal concerns the third.

The brain must continually solve a control problem. It must prevent global activity from collapsing into pathological synchronization, while still allowing large-scale coherence when necessary for sleep, attention, memory, and conscious access. It must let local circuits compute rapidly while enabling slower organism-level state transitions. It must adapt the gain of prediction errors, the thresholds of plasticity, the excitability of cortical populations, and the coupling among distant regions. These functions require slow variables that are distributed enough to shape large-scale dynamics and local enough to interact with synapses and extracellular physiology.

Astrocytes are natural candidates for this role. They are anatomically interposed between synapses, blood vessels, extracellular space, and neuromodulatory inputs. They regulate potassium buffering, neurotransmitter uptake, gliotransmission, lactate availability, vascular coupling, and synaptic plasticity thresholds (Perea et al., 2009; Giaume et al., 2010; Araque et al., 2014; Clasadonte et al., 2017; Reitman et al., 2023). Their dynamics are not fast in the way spikes are fast; this is not a weakness. A control variable should often be slower than the controlled variable. Thermostats do not oscillate at the frequency of the air molecules they regulate. Similarly, a glial control field need not oscillate at theta frequency to tune the neural conditions under which theta organization becomes stable.

This reframes the low-frequency resonome. The resonome is neuronal in its electrical expression but glio-neural in its control architecture. Neural populations generate the measurable electromagnetic currents; astrocytic syncytia modulate the slow constraints within which those currents become organized. The same structural connectome can support different resonome geometries depending on arousal, sleep pressure, metabolic state, neuromodulatory tone, pathology, and developmental history. A complete theory of the resonome should therefore include not only neuronal graphs and cortical fields, but also glial control fields.

## 3 Astrocytic syncytia as mesoscale control substrates

Astrocytes are not passive support cells. They participate in synaptic transmission, circuit state regulation, plasticity, metabolic coupling, extracellular homeostasis, and neurovascular dynamics (Perea et al., 2009; Giaume et al., 2010; Araque et al., 2014; Volterra et al., 2014). At the scale relevant for the present hypothesis, the key property is not merely that individual astrocytes modulate individual synapses. It is that astrocytes are organized into spatially extended, gap-junction-coupled syncytia and domain-like tissue architectures capable of integrating physiological information across local domains and, under some conditions, larger tissue territories (Bushong et al., 2002; Giaume et al., 2010; Ma et al., 2016).

Gap junctions formed by connexins allow astrocytes to exchange ions and small signaling molecules. Astroglial networks mediated by connexins have long been recognized as a major form of glial organization (Giaume et al., 2010; Scemes and Giaume, 2006; Ma et al., 2016). These networks support intercellular calcium and IP3 signaling, potassium redistribution, metabolic substrate trafficking, and coordinated responses to neural activity. Astrocyte topology matters: modeling studies indicate that network architecture can shape propagation, synchrony, and long-distance calcium-wave dynamics (Goldberg et al., 2010; Lallouette et al., 2014; Verisokin et al., 2021). The syncytium is therefore not merely a collection of independent glial cells. It is a coupled physiological substrate.

Astrocytic dynamics are also strongly state-dependent. Nimmerjahn et al. (2009) showed that motor behavior activates Bergmann glial networks. Asada et al. (2015) demonstrated sensory modulation of ongoing astrocytic microdomain calcium dynamics. Poskanzer and Yuste (2016) reported that astrocytes regulate cortical state switching *in vivo*. Reitman et al. (2023) linked norepinephrine, astrocytic activity, and cortical state regulation. These findings make it plausible that astrocytes participate in the slow state variables of the brain, especially those related to arousal, vigilance, and global synchrony.

Astrocytes also regulate synaptic function. The tripartite synapse literature has established that astrocytes sense and modulate synaptic activity through glutamate uptake, gliotransmitter signaling, D-serine, ATP/adenosine pathways, potassium buffering, and receptor-dependent calcium responses (Perea et al., 2009; Araque et al., 2014). Astrocyte signaling can shape spike-timing-dependent depression and plasticity thresholds (Min and Nevian, 2012). Astroglial networks can scale synaptic activity and plasticity (Pannasch et al., 2011). Astrocytic cAMP signaling modulates memory-related plasticity (Zhou et al., 2021). These functions are exactly the functions one expects from a slow gain-control system.

The developmental dimension further strengthens the hypothesis, although it lies mostly beyond the scope of this article. Astrocytic syncytia do not appear fully mature at the outset of neural development; their formation and functional maturation proceed over developmental time (Zhong et al., 2023). This suggests that mature resonome organization may partly reflect the developmental history of glial-neural coupling. A possible developmental extension would investigate whether radial-glial and astrocytic architectures contribute to morphogenetic constraint landscapes that influence later resonome organization. That extension should be treated in a separate paper. Here it is enough to note that the mature low-frequency resonome may inherit constraints from the development of glial syncytial organization.

The biological role proposed here is therefore neither mystical nor merely metaphorical. Astrocytes occupy a unique physiological position. They can integrate slow biochemical, metabolic, ionic, synaptic, and neuromodulatory information. They are distributed across tissue. They interact bidirectionally with neurons. They can alter the excitability and stability of neuronal populations. These properties make them plausible substrates for effective control fields.

## 4 Glial syncytial control fields: minimal mathematical formulation

The goal of this section is not to provide the complete mathematical machinery of the Syncytial Mesh Model. Full graph-to-field derivations, stochastic analysis, numerical schemes, empirical estimators, and null-model comparisons belong to a technical modeling article. The aim here is more modest and more appropriate for a conceptual Frontiers paper: to give enough mathematical structure to make the hypothesis precise, falsifiable, and non-metaphorical.

Let Ω denote a cortical or source-space domain. Let *u*_*g*_(*x, t*) denote an effective glial syncytial control field over Omega. The variable u_g is not microscopic calcium concentration in a single astrocyte. It is a coarse-grained state variable representing the slow control condition of an astrocytic syncytial domain after averaging over many local glia-neuron interactions. It may summarize ionic state, calcium/IP3 excitability, gap-junction coupling, metabolic support, and neuromodulator-sensitive glial responsiveness. The minimal field equation is:


$$\begin{array}{l} \partial_{t}^{2}u_{g}\left(x,t\right)+\Gamma_{g}\left(x,t\right)\partial_{t}u_{g}\left(x,t\right)\\ =\nabla \cdot \left(D_{g}\left(x,t\right)\nabla u_{g}\left(x,t\right)\right)+S_{n}\left(x,t\right)+\eta_{g}\left(x,t\right). \end{array}$$

Here *D*_*g*_ is an effective spatial coupling tensor, Γ_*g*_ is an effective damping term, S_n is neuronal forcing of the astrocytic layer, and eta_g summarizes stochastic physiological fluctuations. The equation is intentionally effective. It does not claim that astrocytes literally support linear waves with the same speed or frequency as neuronal EEG rhythms. It states that, after coarse-graining, the astrocytic syncytium contributes slow spatially coupled control dynamics. Accordingly, the displayed second-order equations define an effective control-field ansatz, not the unique microscopic dynamics of the astrocytic layer; channel-specific models may instead use first-order reaction-diffusion, graph-diffusion, or other coarse-grained variants when these are experimentally or computationally appropriate. Likewise, *D*_*g*_, Γ_*g*_, *G*_*g*_, and Π_*g*_ are inferential control parameters to be identified through perturbational data and model-comparison procedures, not direct cellular measurements.

The corresponding low-frequency neuronal electromagnetic field variable is written ψ_*LF*_(*x, t*). This variable represents the neuronal expression of low-frequency EM organization: the kind of activity that, after source projection and volume conduction, becomes visible in EEG, MEG, ECoG, or local field potentials. The glial field affects ψ_*LF*_ by changing the parameters of neuronal dynamics rather than by replacing neuronal current generation. A compact expression is:


$$\begin{array}{l} \partial_{t}^{2}\psi_{LF}\left(x,t\right)+\Gamma_{g}u_{g},m\partial_{t}\psi_{LF}\left(x,t\right)\\ =L_{g}u_{g},m\psi_{LF}\left(x,t\right)+I_{n}\left(x,t\right)+\xi_{n}\left(x,t\right), \end{array}$$

where *I*_*n*_ denotes neuronal current generation, *m*(*t*) denotes neuromodulatory state variables, Γ_*g*_[*u*_*g*_, *m*] is glial-controlled damping, and $L_{g}\left[u_{g},m\right]$ is a glial-constrained spatial coupling or gain operator. This equation expresses the central thesis in mathematical form: the measured resonome is neuronal in its electrical expression but glio-neural in its control geometry.

The modal structure follows immediately. Let the effective glial spatial operator be


$$\begin{array}{l} A_{g}u=-\nabla \cdot \left(D_{g}\nabla u\right), \end{array}$$

with eigenfunctions ϕ_*k*_ and eigenvalues λ_*k*_, so that *A*_*g*_ϕ_*k*_ = λ_*k*_ϕ_*k*_. Expanding the glial field as


$$\begin{array}{l} u_{g}\left(x,t\right)=\underset{k}{\sum }a_{k}\left(t\right)\phi_{k}\left(x\right), \end{array}$$

yields, in the constant-damping approximation,


$$\begin{array}{l} {ä}_{k}\left(t\right)+\Gamma_{g}{ȧ}_{k}\left(t\right)+\lambda_{k}a_{k}\left(t\right)=s_{k}\left(t\right)+\eta_{k}\left(t\right). \end{array}$$

Each spatial mode therefore behaves as a damped driven oscillator. Low spatial modes have broad support and are more likely to persist under damping and heterogeneity. High spatial modes are more vulnerable to dissipation, local irregularity, and boundary effects. This gives a principled reason why slow, large-scale organization should be especially sensitive to glial control: low-frequency resonome structure is precisely the regime in which broad spatial modes and slow damping parameters matter most.

The same framework can be written in state-space form. Let X(t) denote the state of relevant neuronal populations and field variables. Then:


$$\begin{array}{l} Ẋ=F\left(X;\Theta_{g},m\right)+\xi ,\;\;\Theta_{g}=\left(D_{g},\Gamma_{g},G_{g},\Pi_{g}\right). \end{array}$$

The parameter tuple Θ_*g*_ represents glial control over spatial coupling *D*_*g*_, damping Γ_*g*_, gain *G*_*g*_, and precision or salience weighting Π_*g*_. The neuromodulatory state m changes glial responsiveness. This formulation provides a bridge to predictive processing and active inference: if neuronal inference depends on precision weighting of prediction errors, then astrocytic syncytia may contribute to the slow regulation of precision geometry. In schematic terms:


$$\begin{array}{l} \Pi \to \Pi_{g}\left(u_{g},m\right), \end{array}$$

meaning that prediction errors may be amplified, suppressed, stabilized, or ignored depending partly on the glial control state. This does not reduce active inference to astrocytes. It adds a plausible biological substrate for slow precision modulation.

In empirical applications, *D*_*g*_, Γ_*g*_, *G*_*g*_, and Π_*g*_ would not initially be inferred as literal microscopic astrocytic quantities. They would be operationalized through measurable field-level observables: coherence decay length as a proxy for effective spatial coupling, phase-gradient persistence and wave-vector autocorrelation as proxies for low-mode stability, damping estimates from perturbation recovery or state transitions, gain changes from stimulus-response or state-dependent amplification, and precision-like weighting from changes in prediction-error amplification or attentional and arousal modulation. The model is therefore testable by asking whether astrocyte-specific manipulations explain variance in these observables beyond neuron-only, connectome-only, vascular, or phase-randomized null models.

**Figure 2:**
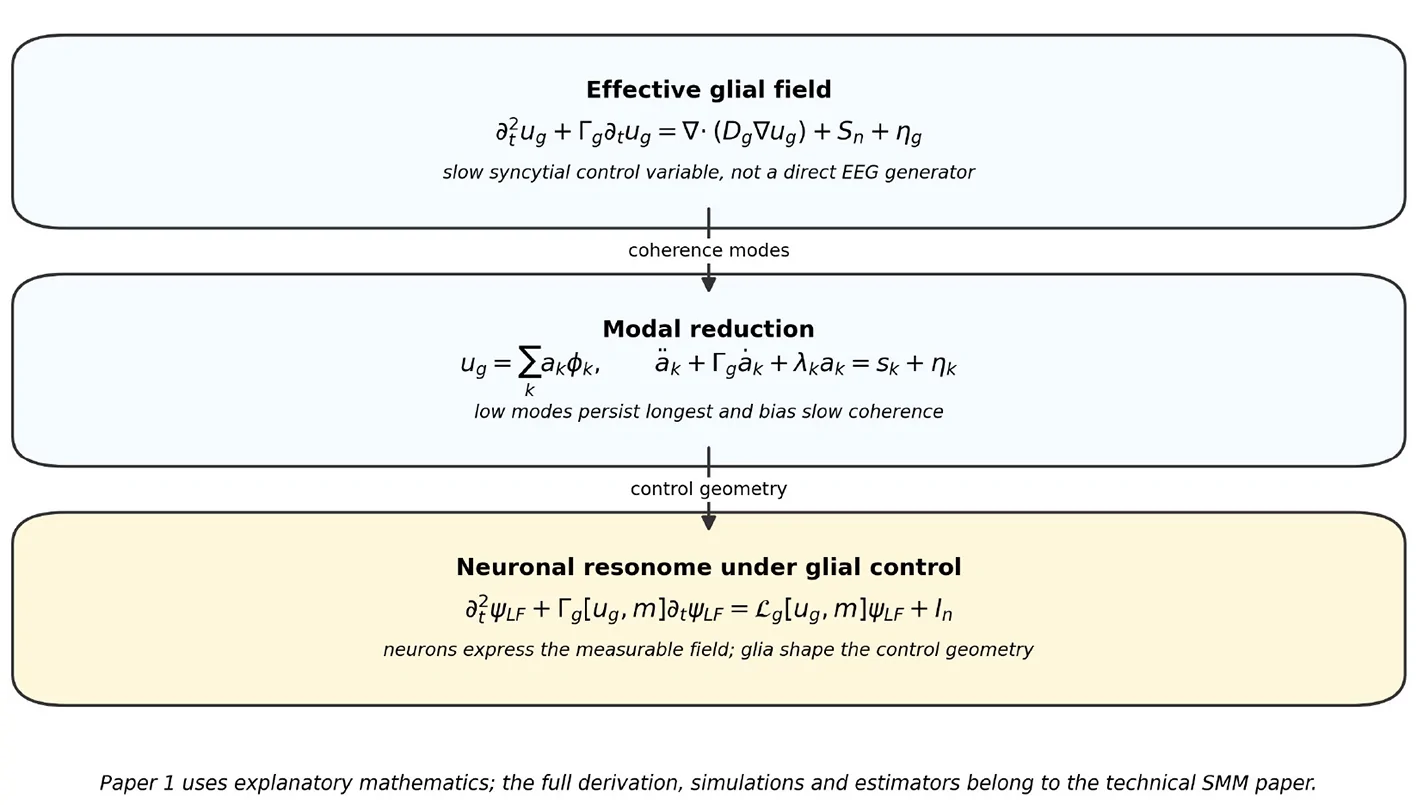
Minimal mathematical spine of the hypothesis. Paper 1 uses explanatory mathematics only. The glial field *u*_*g*_ is an effective slow control variable; its low modes bias coherence and damping; the neuronal low-frequency resonome ψ_*LF*_ remains the source of the measurable field expression. Detailed derivations, numerical simulations, parameter inference, and empirical estimators belong to the technical SMM paper.

The mathematical relations are summarized schematically in Figure 2.

This minimal mathematics also clarifies what the model is not. It is not a claim that astrocytic calcium waves directly travel across centimeters at delta/theta frequencies. It is not a claim that astrocytes are the dominant EEG/MEG current source. It is not a claim that electromagnetic fields alone constitute consciousness. The model is a graph-field-control hypothesis: astrocytic syncytial organization supplies slow control parameters that shape the neuronal resonome.

## 5 Four physiological channels of glial control

A theory of glial syncytial control fields must specify how the field acts. Otherwise it risks becoming a metaphor. I propose four interacting physiological channels: ionic/extracellular control, synaptic-gain and metaplastic control, metabolic-neurovascular control, and neuromodulatory precision/state control. These are functional control classes rather than mutually exclusive molecular mechanisms. For that reason, gliotransmission is treated under synaptic-gain and metaplastic control, not because it is biologically secondary, but because in the present control-theoretic decomposition its main effect is on synaptic efficacy, plasticity thresholds, and gain transfer from local activity to large-scale field organization. Each channel is grounded in known astrocyte biology; the novelty lies in treating them together as components of a mesoscale control architecture.

**Figure 3:**
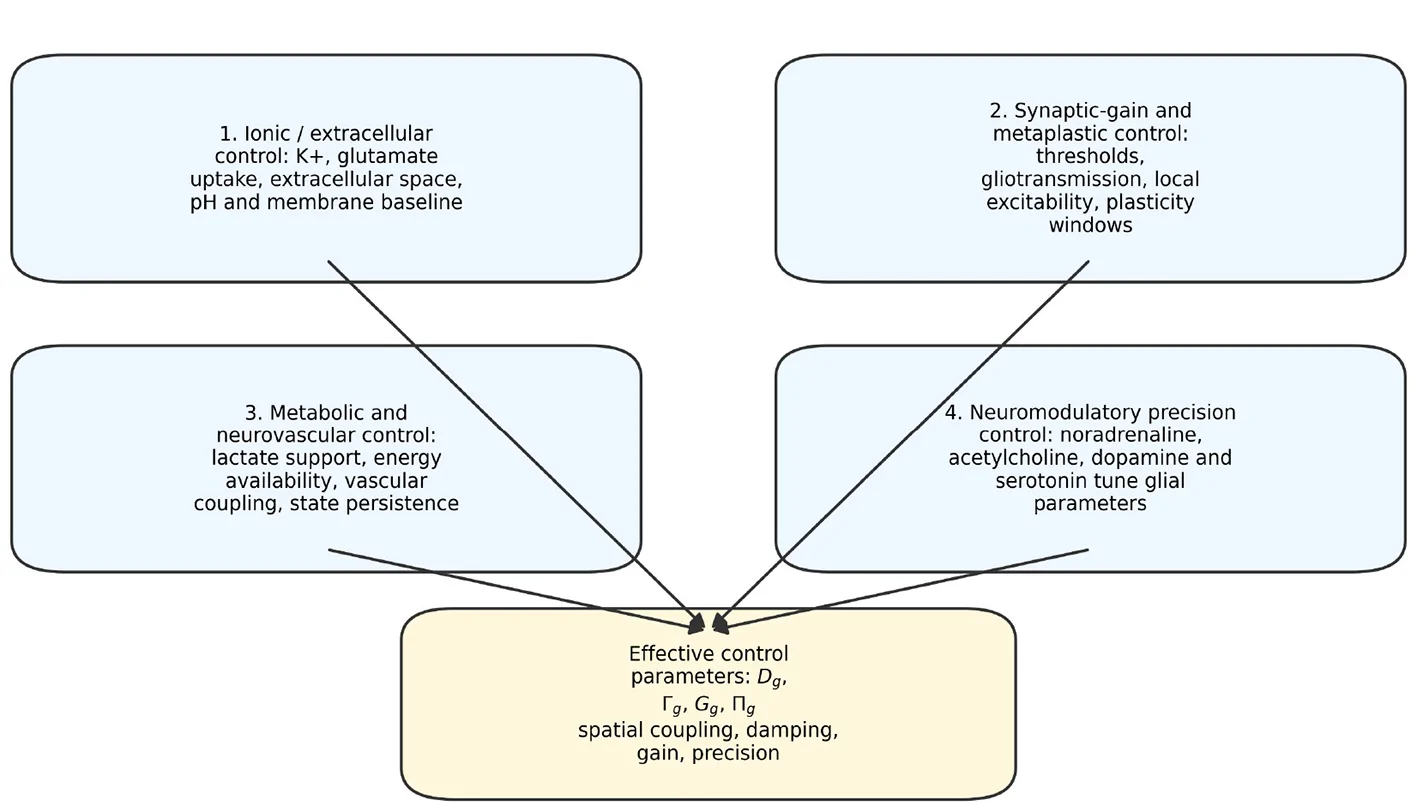
Four physiological channels of glial control. Astrocytic syncytia can influence low-frequency neuronal field organization through ionic/extracellular regulation, synaptic-gain and metaplastic regulation, metabolic-neurovascular regulation, and neuromodulatory precision/state regulation. These channels jointly determine spatial coupling, damping, gain, and precision-control parameters.

These four channels are illustrated in Figure 3.

### 5.1 Ionic and extracellular control

Neuronal excitability depends on extracellular ionic conditions. Astrocytes regulate extracellular potassium, neurotransmitter uptake, water balance, extracellular space, and pH (Perea et al., 2009; Giaume et al., 2010; Pannasch et al., 2011). These factors directly affect membrane potential, local excitability, synaptic efficacy, and the susceptibility of neuronal populations to synchronize. A glial syncytium coupled through gap junctions can spatially redistribute ionic and metabolic loads, thereby changing the effective damping and excitability landscape of neuronal tissue.

Within the mathematical formulation, ionic/extracellular control primarily modifies Γ_*g*_, *G*_*g*_, and components of *L*_*g*_. Increased extracellular potassium or impaired uptake can move local circuits closer to synchronization or pathological excitability. Stronger buffering can damp excessive fluctuations. Because such regulation is spatially extended through astrocytic networks, it is a natural mechanism for influencing traveling-wave persistence and low-frequency coherence.

### 5.2 Synaptic-gain and metaplastic control

Astrocytes sense synaptic activity and modulate synaptic transmission through neurotransmitter uptake, receptor-dependent signaling, gliotransmission, and plasticity-related pathways (Perea et al., 2009; Araque et al., 2014). They influence not only immediate synaptic efficacy but also the thresholds and windows within which plasticity occurs (Min and Nevian, 2012; Pannasch et al., 2011; Zhou et al., 2021). This is why astrocytes are especially relevant to low-frequency resonome dynamics: slow field structure is not merely an expression of current activity, but also a regulator of which activity patterns become stabilized over time.

In the control-field formulation, synaptic-gain and metaplastic control alter *G*_*g*_ and Π_*g*_. The glial field changes the gain with which local neuronal activity contributes to large-scale organization. It can thereby bias the system toward desynchronization, local processing, global integration, slow oscillation, or pathological hypersynchrony. The same neuronal input may produce different resonome consequences under different glial control states.

### 5.3 Metabolic and neurovascular control

Large-scale low-frequency activity is energetically expensive and state-dependent. Astrocytes participate in metabolic support, lactate shuttling, blood-flow regulation, and neurovascular coupling (Giaume et al., 2010; Clasadonte et al., 2017; Reitman et al., 2023). These processes unfold over slower timescales than spikes and fast synaptic events. They are therefore well positioned to shape the persistence and recoverability of low-frequency electromagnetic organization.

Metabolic-neurovascular control affects the resonome by modulating the capacity of tissue to sustain coherent activity. In mathematical terms, it modifies damping, recovery time constants, and the viability of persistent modes. A slow wave or phase-gradient structure that is metabolically unsupported will dissipate; a structure supported by glial metabolic coupling may persist. This provides a physiological path from astrocytic state to the stability of slow resonome organization.

### 5.4 Neuromodulatory precision and state control

Astrocytes respond strongly to neuromodulators such as noradrenaline and acetylcholine, and interact with dopamine and serotonin systems in state-dependent ways (Poskanzer and Yuste, 2016; Reitman et al., 2023). Neuromodulators are not merely chemical background variables; they tune global state, arousal, attention, uncertainty, salience, and sleep-wake transitions. Astrocytes may act as integrators and transducers of these signals into slow changes in excitability, gain, and coupling.

Let m(t) denote neuromodulatory state. Then:


$$\begin{array}{l} D_{g}=D_{g}\left(m\right),\Gamma_{g}=\Gamma_{g}\left(m\right),G_{g}=G_{g}\left(m\right),\Pi_{g}=\Pi_{g}\left(m,u_{g}\right). \end{array}$$

The same anatomical connectome can therefore support different resonome structures under different neuromodulatory conditions. This provides a direct mechanistic bridge between glial syncytial control fields and global conscious state. Wakefulness, drowsiness, sleep, anesthesia, delirium, and focused attention need not differ only in neuronal firing patterns; they may differ in the glial control parameters that determine which neuronal electromagnetic modes become stable.

## 6 Relation to existing theories

The glial syncytial control-field hypothesis is not intended to replace existing theories of large-scale brain dynamics. It adds a biological control layer to frameworks that already explain important parts of the resonome through neural-field propagation, connectome geometry, predictive processing, active inference, electromagnetic field organization, resonance, and global workspace dynamics.

### 6.1 Neural-field and connectome models

Neural-field and connectome models correctly identify major constraints on propagation, pattern formation, and large-scale modal structure (Atasoy et al., 2016; Muller et al., 2018). The present hypothesis keeps those constraints intact but places them inside a glial control environment. Structural connectivity specifies possible routes of neuronal influence; glial syncytial control fields are proposed to modulate the gain, damping, persistence, and state-dependent accessibility of those routes. Thus connectome harmonics describe available neuronal resonances, whereas glial control fields contribute to the conditions under which particular harmonics become amplified, suppressed, stabilized, or metastable.

This distinction also clarifies the relation to recent glia-inclusive models. Ali et al. (2025) and Handy and Borisyuk (2023) show that astrocytic mechanisms can affect large-scale fast-slow fluctuations, phase-amplitude relations, and network synchrony. The present proposal is narrower in one respect and stronger in another: it does not claim that astrocytes are the primary generators of the measured field, but it formalizes astrocytic syncytia as continuous effective control variables acting on low-frequency field geometry.

### 6.2 Predictive processing and active inference

Predictive processing and active inference require biological mechanisms for precision weighting (Friston, 2010). Neuromodulatory systems are usually invoked, but a tissue-level mechanism must also integrate neuromodulatory tone with local excitability, extracellular state, synaptic thresholds, metabolic availability, and large-scale coherence. Glial syncytial control fields offer one such implementation layer. Astrocytes can integrate neuromodulatory, synaptic, extracellular, and metabolic signals over slow windows; in doing so, they may contribute to the precision landscape determining which prediction errors are amplified, suppressed, or stabilized. This does not make glia inferential agents in isolation; it assigns them a plausible role in the slow control of neuronal inference dynamics.

### 6.3 Electromagnetic, resonance, and workspace theories of consciousness

Electromagnetic and resonance theories emphasize structured field organization and nested synchronization, while global workspace theories emphasize ignition, broadcast, and widespread availability (Dehaene and Changeux, 2011; Hunt and Schooler, 2019; McFadden, 2020). The present framework is compatible with these approaches but keeps a conservative causal distinction: astrocytes are not proposed to generate the conscious electromagnetic field directly. They are proposed to constrain the neuronal low-frequency patterns that may support availability, coherence, and state transitions. In workspace terms, the glial control field contributes not content but readiness conditions: gain, damping, and stability parameters that help determine whether local neuronal activity remains local, becomes globally available, or becomes pathologically hypersynchronous.

## 7 Empirical predictions

The value of a glial control-field theory depends on discriminative predictions. Some predictions below overlap with known general astrocytic effects on oscillations, synchrony, sleep, and cortical state. They are included because they define the empirical domain of the hypothesis. The discriminative predictions of the model concern not mere changes in power or synchrony, but changes in the geometry, persistence, damping, coherence length, low-mode dominance, and state-dependence of source-space low-frequency fields. The model must therefore be tested against neuron-only, connectome-only, vascular-only, and phase-randomized alternatives.

**Figure 4:**
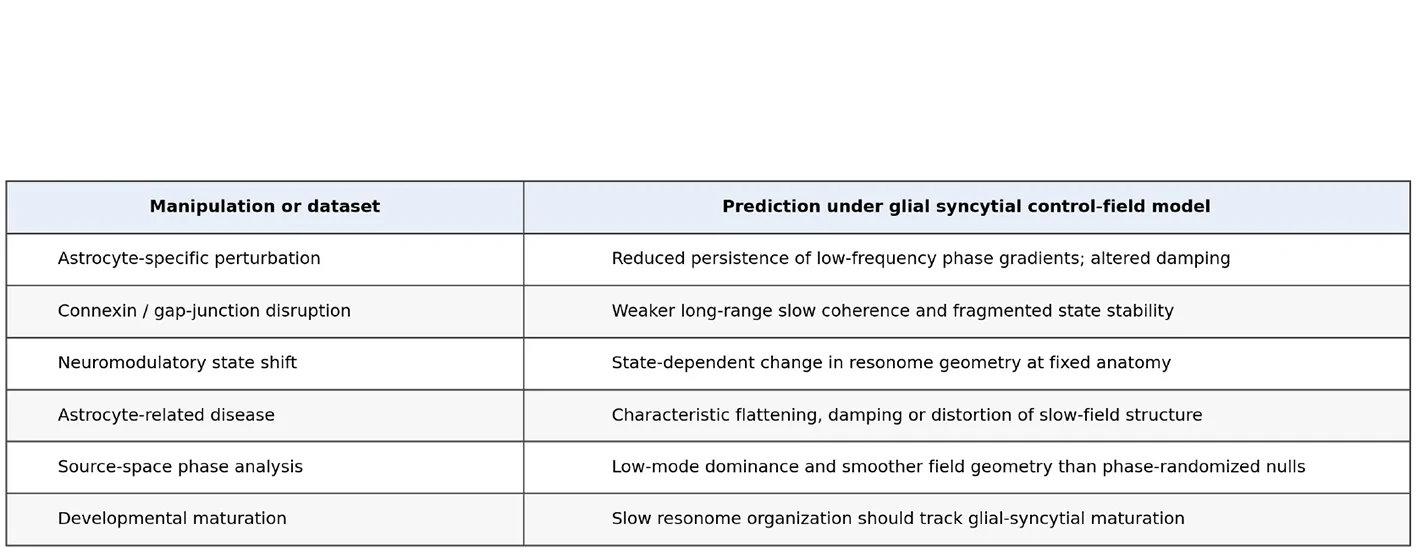
Falsifiable prediction space. The glial syncytial control-field hypothesis predicts that astrocyte-specific perturbation, gap-junction disruption, neuromodulatory state shifts, astrocyte-related pathology, source-space phase analysis, and developmental maturation should produce characteristic changes in low-frequency resonome structure.

The resulting prediction space is summarized in Figure 4.

### 7.1 Astrocyte-specific perturbation should alter slow cortical synchrony

If astrocytic syncytia contribute to low-frequency control geometry, then astrocyte-specific perturbations should alter slow cortical synchronization, state stability, and traveling-wave persistence even when direct neuronal stimulation is held constant. Existing work already points in this direction: astrocytic signaling participates in cortical state switching, norepinephrine-linked astrocytic activity is involved in state-dependent resynchronization, and astrocyte connexin disruption changes sleep-wake architecture (Poskanzer and Yuste, 2016; Reitman et al., 2023; Clasadonte et al., 2017). Future experiments should combine astrocyte-specific optogenetic, chemogenetic, pharmacological, or genetic manipulations with high-density EEG/MEG/ECoG source reconstruction.

The prediction is not simply that neural activity changes. Any perturbation of brain tissue may change neural activity. The specific prediction is that glial perturbation should change the **geometry and persistence** of low-frequency field organization: phase-gradient duration, low-mode dominance, damping estimates, traveling-wave coherence, and state-transition thresholds.

### 7.2 Gap-junction modulation should change coherence length and damping

Connexin-mediated coupling is central to astrocytic syncytial organization. The model predicts that disrupting astrocytic gap-junction coupling should reduce long-range low-frequency coherence, fragment the spatial smoothness of phase fields, and increase effective damping of slow resonome modes. Conversely, conditions that enhance functional astrocytic coupling should increase coherence persistence, within physiological limits.

This prediction is especially discriminative because neuron-only models do not naturally predict that astrocyte-specific gap-junction changes should alter low-frequency field geometry unless glia are included as causal control variables. The expected effect is not necessarily a simple reduction in power. It may appear as reduced spatial smoothness, shorter phase-gradient persistence, altered coherence decay length, or increased variability of state transitions.

### 7.3 Traveling-wave persistence should exceed phase-randomized and neuron-only nulls

The strongest empirical tests are likely geometric rather than purely spectral. A glial control-field model predicts that low-frequency phase gradients should persist smoothly over time and space more than expected under phase-randomized surrogates, local neural-noise models, or connectome-only null models. The relevant observable is not merely whether theta power exists, but whether the phase field behaves like a low-dimensional damped field.

A source-space analysis could compute phase-gradient persistence time, wave-vector autocorrelation, orientation entropy, curvature statistics, and coherence decay. If empirical slow-wave geometry is smoother and more persistent than neuron-only nulls predict, and if this persistence is reduced by astrocyte-specific perturbation or glial pathology, the evidence for glial control fields would be strong.

### 7.4 Low-dimensional resonome structure should track state and pathology

The model predicts that low-frequency resonome dynamics should be low-dimensional in source space because low spatial modes survive damping more readily than high modes. This does not imply sharp harmonic resonances at fixed frequencies across all subjects. Biological tissue is heterogeneous, damped, noisy, and state-dependent. The prediction is broader: after appropriate source reconstruction and detrending, slow field organization should lie near a small number of smooth modes whose geometry changes with glial state.

Astrocyte-related disorders should therefore alter low-frequency resonome geometry. Candidate conditions include epilepsy, Alzheimer's disease, traumatic brain injury, sleep disorders, major depression, neurodevelopmental disorders, and gliopathies. The expected signatures include altered damping, coherence decay, low-mode variance, traveling-wave persistence, and state-transition stability. These biomarkers would be more specific than broadband power changes.

### 7.5 Neuromodulatory manipulation should change resonome geometry at fixed anatomy

Because astrocytes respond to neuromodulators, the model predicts that arousal and neuromodulatory manipulation should change the geometry of the low-frequency resonome even when anatomy is unchanged. Pharmacological or behavioral manipulations of noradrenergic, cholinergic, dopaminergic, or serotonergic tone should alter glial control parameters and therefore change slow field damping, gain, and coherence persistence.

This provides an explanatory bridge between global state and field geometry. The same brain under quiet wakefulness, focused attention, drowsiness, and sleep may support different resonome structures because the glial control parameters differ. This claim can be tested by combining neuromodulatory manipulations, pupil/arousal measures, EEG/MEG source analysis, and ideally astrocyte-sensitive imaging or biomarkers.

### 7.6 Developmental prediction

A developmental extension follows naturally. If glial syncytial organization contributes to mature low-frequency control geometry, then the maturation of slow resonome structure should track the maturation of astrocytic syncytial organization, not only synaptogenesis or white-matter connectivity. During development, radial-glial and astrocytic architectures may help shape the constraints within which neuronal circuits stabilize. Mature resonome geometry may therefore partly reflect developmental canalization by glial morphogenetic architecture.

This claim is intentionally reserved for future work. It is stronger and more speculative than the mature-brain control-field hypothesis. It should be developed with developmental data, radial-glial biology, morphogenetic modeling, and longitudinal recordings. The present paper only marks it as a prediction space.

## 8 Falsifiability and limitations

The hypothesis is falsifiable. It would be weakened if adequately powered astrocyte-specific perturbations left source-space low-frequency phase geometry unchanged; if neuron-only, connectome-only, vascular, or phase-randomized null models explained traveling-wave persistence, damping, and low-mode structure with equal or greater parsimony; if estimated damping, gain, or coherence parameters showed no relation to astrocytic physiological state; or if glial pathology produced no systematic alteration of slow resonome organization.

Several limitations follow. First, the model is an effective mesoscale theory: *u*_*g*_ is a coarse-grained control variable, not a direct measurement of one microscopic astrocytic quantity. Second, astrocytes are not proposed to be the dominant generators of EEG or MEG currents; the principal extracranial generators remain organized neuronal synaptic currents, especially in pyramidal populations. Third, the model does not claim that consciousness is reducible to astrocytes, electromagnetic fields, or low-frequency oscillations. It claims only that glial syncytial control fields may help stabilize the low-frequency resonome within which some conscious-state dynamics unfold.

Finally, existing evidence is suggestive but not decisive, and the framework is compatible with causal redundancy. Structural connectivity, cortical geometry, synaptic delays, thalamocortical loops, neuromodulation, vascular dynamics, and body-state signals all contribute to low-frequency organization. The hypothesis is important not because it replaces these factors, but because it identifies a specific, testable glial control layer that may interact with all of them.

## 9 Research program

A mature research program should proceed along four lines. First, it should prioritize field-level observables rather than scalp power alone: source-space phase geometry, traveling-wave persistence, low-mode dominance, damping estimates, coherence decay functions, wave-vector autocorrelation, and state-transition thresholds. Second, it should combine field-level recordings with direct astrocyte manipulations, including optogenetic, chemogenetic, pharmacological, genetic, connexin-related, calcium-pathway, neuromodulatory, and metabolic interventions. Third, it should compare glial control-field models against strong alternatives: neural-field models, connectome-only models, Kuramoto-type models, vascular confounds, and phase-randomized nulls. Fourth, a technical companion paper should derive the effective field from astrocyte-neuron graph dynamics, define inference procedures for *D*_*g*_, Γ_*g*_, *G*_*g*_, and Π_*g*_, simulate realistic cortical manifolds, test source-space predictions, and report code and data transparently.

A further extension concerns primate and human astrocyte geometry. Human astrocytes show distinctive morphological complexity and cellular organization compared with commonly studied rodent preparations (Oberheim et al., 2006, 2009). If some primate- or human-enriched astrocyte populations depart from simple domain tiling, the control-field framework predicts that their unusual geometry should matter most where large-scale integration, flexible state control, and higher cognitive availability require nonlocal coordination. This point is speculative and is not needed for the present mature-brain control hypothesis, but it defines a natural comparative and developmental extension of the model.

If distributed astrocytic state variables systematically modulate neuronal gain, damping, coupling, precision weighting, or stability over timescales longer than dominant neuronal oscillations, then astrocytic syncytia constitute an effective control layer for low-frequency resonome dynamics.

## 10 Conclusion

The low-frequency resonome is where sleep, arousal, large-scale coordination, pathology, and conscious access become visible as structured electromagnetic organization. Existing neuron-centered and glia-inclusive theories explain important parts of this structure, but the specific slow physiological control substrate that tunes its damping, gain, coupling, and stability remains incompletely specified.

This article has proposed that astrocytic syncytia may provide such a substrate. Through gap-junction coupling, calcium/IP3 signaling, extracellular ionic regulation, synaptic-gain modulation, metabolic support, neurovascular interaction, and neuromodulatory responsiveness, astrocytes are well positioned to generate effective mesoscale control fields. These fields do not replace neuronal electromagnetic generation; they shape the control geometry within which neuronal electromagnetic organization becomes stable, metastable, desynchronized, or pathological.

The compact thesis is:

**astrocytic syncytium -> slow glial control field -> damping, gain, coupling, precision -> low-frequency neuronal resonome**.

If correct, this framework changes the ontology of large-scale brain dynamics. The brain should not be modeled only as synchronization over a neuronal graph or propagation over a neural field. It should also be modeled as the interaction between neuronal current-generating systems and slow glial syncytial control fields.

## Data Availability

The original contributions presented in the study are included in the article/supplementary material, further inquiries can be directed to the corresponding author.
